# Cloning and characterization of a tyrosine decarboxylase involved in the biosynthesis of galanthamine in *Lycoris aurea*

**DOI:** 10.7717/peerj.6729

**Published:** 2019-04-16

**Authors:** Rong Wang, Xiaokang Han, Sheng Xu, Bing Xia, Yumei Jiang, Yong Xue, Ren Wang

**Affiliations:** 1 Jiangsu Key Laboratory for the Research and Utilization of Plant Resources, Institute of Botany, Jiangsu Province and Chinese Academy of Sciences, Nanjing, China; 2 Eco-environmental Protection Research Institute, Shanghai Environmental Protection Monitoring Station of Agriculture, Shanghai Engineering Research Centre of Low-carbon Agriculture (SERLA), Shanghai Key Laboratory of Protected Horticultural Technology, Shanghai Academy of Agricultural Sciences, Shanghai, China

**Keywords:** Tyrosine decarboxylase, Amarylidaceae alkaloids, *Lycoris aurea*, Galanthamine

## Abstract

**Background:**

Galanthamine, one kind of Amaryllidaceae alkaloid extracted from the *Lycoris* species, is used in the treatment of Alzheimer’s disease. In regards to medical and economic importance, the biosynthesis and regulatory mechanism of the secondary metabolites in *Lycoris* remain uninvestigated.

**Methods:**

BLAST was used to identify the sequence of tyrosine decarboxylase in the transcriptome of *Lycoris aurea* (L’Hér) Herb. The enzyme activity of this TYDC was determined by using heterologous expressed protein in the *Escherichia coli* cells. The related productive contents of tyramine were detected using High Performance Liquid Chromatography (HPLC). According to the available micro RNA sequencing profiles and degradome database of *L. aurea*, microRNA396 were isolated, which targets to *LaTYDC1* and RNA Ligase-Mediated-Rapid Amplification of cDNA Ends (RLM-RACE) were used to confirm the cleavage. The expression levels of miR396 and *LaTYDC1* were measured using a quantitative real-time polymerase chain reaction (qRT-PCR).

**Results:**

LaTYDC1 was mainly expressed in root, bulb, leaf and flower fitting the models for galanthamine accumulation. This decarboxylase efficiently catalyzes tyrosine to tyramine conversion. Under methyl jasmonate (MeJA) treatment, the expression of *LaTYDC1* and the content of tyramine sharply increase. The use of RLM-RACE confirms that miR396 promotes the degradation of *LaTYDC1* mRNA. Under MeJA treatment, the expression of miR396 was suppressed while the expression level of *LaTYDC1* sharply increased. Following the increase of the miR396 transcriptional level, *LaTYDC1* was significantly repressed.

**Conclusion:**

LaTYDC1 participates in the biosynthesis of galanthamine, and is regulated by miR396. This finding also provides genetic strategy for improving the yield of galanthamine in the future.

## Introduction

Plants generate and accumulate secondary metabolites in response to a wide variety of biotic and abiotic stresses, particularly in defense against herbivores or pathogens (Kabera et al., 2014; Yu & De Luca, 2013). These secondary metabolites have developed as resources of natural drugs because of their biological activities. Many others have become commercially important and notably beneficial in the treatment of human diseases (Yu & De Luca, 2013). For example, galanthamine, which is extracted from the *Lycoris* plant, is one type of Amaryllidaceae alkaloid. As an inhibitor of cholinesterase, galanthamine can increase acetylcholine sensitivity and has a positive effect when treating Alzheimer’s disease (Dal-Bianco et al., 1991). Other Amaryllidaceae alkaloids, such as lycorine and haemanthamine, have anti-cancer, anti-viral, and anti-bacterial properties. Lycorine might be a good candidate for therapeutic agent against leukemia and severe acute respiratory syndrome (SARS) (Li et al., 2015; Doskočil et al., 2015; Havelek et al., 2014). The officinal and economic values of *Lycoris* species remain uninvestigated.

Since the biosynthetic pathways of Amaryllidaceae were first proposed by Barton, Kirby & Thomas (1963), a few enzymes taking part in the galanthamine synthesis pathway have been identified and characterized (Eichhorn et al., 1998; Kilgore et al., 2014; Kilgore et al., 2016). At the upstream of the galanthamine biosynthesis pathway, tyrosine, which is the origin beginning of isoquinoline alkaloid biosynthesis is converted into tyramine by an undefined tyrosine decarboxylase (TYDC), which is the first committed step in isoquinoline alkaloid biosynthesis. TYDC is a common enzyme in plant kingdom (Lehmann & Pollmann, 2009), and also is implicated in the defense response (Facchini & De Luca, 1994). Then, tyramine and norbelladine 3,4-dihyroxybenzaldehyde are condensed to a Schiff-base (Eichhorn et al., 1998; Kilgore et al., 2014, Fig. 1), which is documented to be the basic bone of all Amaryllidaceae alkaloids (Barton et al., 1961). Norbelladine is then methylated by 4′-*O*-methyltransferase (*Np* N4OMT) to 4′-*O*-methylnorbelladine (Kilgore et al., 2014). One key phenol-coupling enzyme cytochrome P450, CYP96T1 from *Narcissus* sp. aff. *Pseudonarcissus* was identified to have *C-C* phenol coupling capacity to catalyze 4′-*O*-methylnorbelladine to noroxomaritidine (Kilgore et al., 2016).

**Figure 1:**
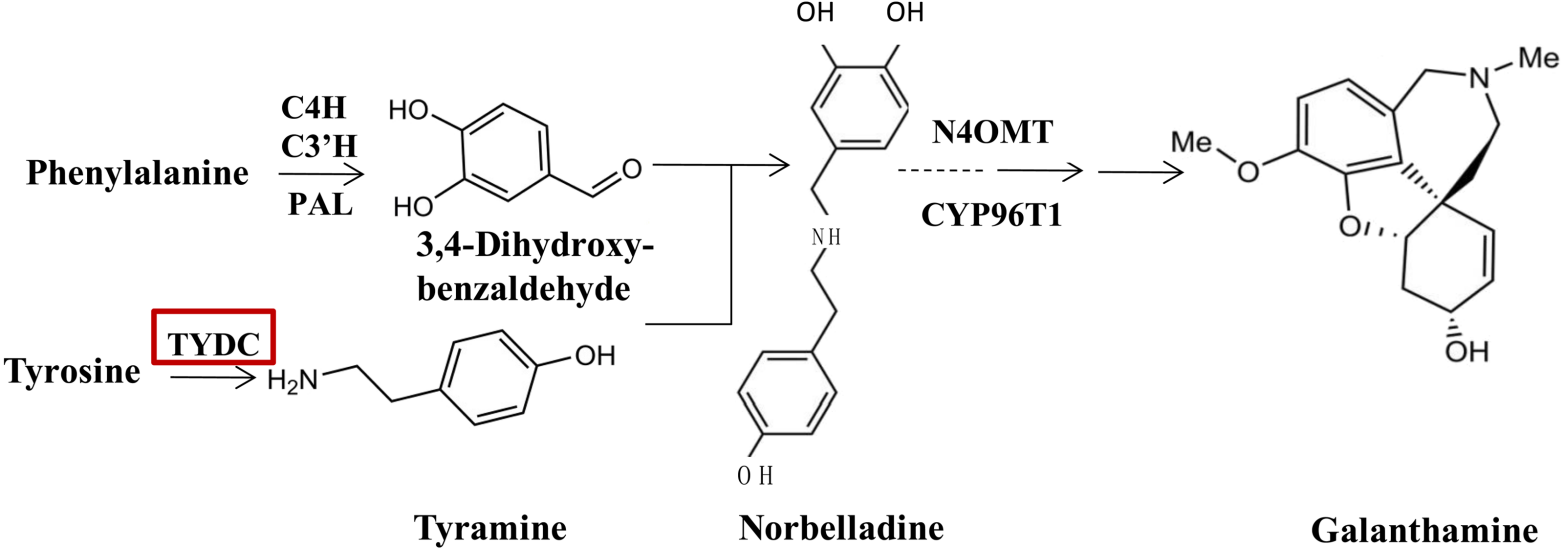
TYDC is involved in the proposed biosynthetic pathway for galanthamine. The enzymes and compounds involved in the galanthamine biosynthetic pathway. PAL, phenylalanin ammo-nialyase; OMT, methyltransferase; CYP, cytochrome P450; C4H, Trans-cinnamic acid 4-hydroxylase; C3′H: Trans-cinnamic acid 3′-hydroxylase.

Jasmonate (JA) signal regulates plant response to many biotic and abiotic stresses by coordinating the production of abundant of defense-related protein and secondary metabolites (Turner, Ellis & Devoto, 2002; Wasternack, 2015). Methyl jasmonate (MeJA) can induce three major classes of plant secondary metabolites (Wasternack, 2007; Wasternack & Hause, 2013). In our observation, under MeJA treatment, the accumulation of galanthamine is induced distinctly in other *Lycoris* plants (Mu et al., 2009); moreover, some specific genes correlated with secondary metabolism respond to MeJA signaling (Farmer & Ryan, 1992). MicroRNAs (miRNA) are commonly found in both plants and animals, these small regulatory RNAs with 20–24 nucleotides have been recognized for their participation in regulation mechanisms in plant growth, development and defense (Chen, 2009). As a post-transcriptional regulator, miRNAs in complex with Argonaute (AGO) effector protein, they cause target mRNA degradation through complementary region binding, while negatively modulating the expression of a wide range of genes. The plant miRNAs regulate their target mRNA by perfectly or near-perfectly complementary-matching (Axtell & Bowman, 2008; Chen, 2009); therefore, based on sequence complementarity, the target of the plant miRNAs can usually be successfully predicted (Sunkar & Zhu, 2004). It is predicted that miRNAs elicited by MeJA correlate with galanthamine biosynthesis via a possible post-transcriptional regulation (Xu et al., 2016). When investigating the role of miRNAs in the regulation of galanthamine biosynthesis in our earlier study, we observed and profiled *L. aurea* miRNA in response to treatment with MeJA. 154 miRNAs expressing differently in comparison to the vehicle-treated ones were identified (Xu et al., 2016). By using degradome sequencing analysis, 32 genes from different plant processes were validated, and were then targeted by 49 miRNAs. Among these miRNAs and targets, we found that LaTYDC1 was targeted by miR396. Of the conserved miRNAs in plants, miR396 regulates the expression level of transcriptional factors, such as GROWTH-REGULATING FACTOR (GRF) and bHLH74, thereby affecting plant growth and development (Debernardi et al., 2014; Liang et al., 2014; Rodriguez et al., 2015).

In this study, we have identified an enzyme called LaTYDC1, which play role in tyrosine to tyramine conversion step of galanthamine biosynthetic pathway in *L. aurea*. Additionally, the expression level of LaTYDC1 and the content of tyramine were regulated by MeJA. Our results also showed that miR396 may act as a negative regulator that promotes the degradation of *LaTYDC1* to repress the yield of tyramine.

## Materials and Methods

### Plant materials and growth conditions

*L. aurea* were planted in a nursery at the Institute of Botany, Jiangsu Province and Chinese Academy of Sciences, Nanjing, China. The flowers and scapes of *L. aurea* were collected in August while bulbs, roots, seeds, and leaves of *L. aurea* were collected in October. For the treatment experiments, the seeds of *L. aurea* were planted in an incubator with 16/8 h (22/18 °C) day/night regimes at 100 µmol m^−2^s^−1^ irradiation. Eight month-old seedlings were imposed for 0, 6, 12 and 24 h with 100 µM MeJA in 0.02% DMSO, or with 0.02% Dimethyl sulfoxide (DMSO) as the vehicle control, respectively, as previously described by Ma et al. (2016). Then each treated sample was collected for further analyses.

### Isolation and cloning of *LaTYDC1* cDNA

By BLAST against the transcriptome database of *L. aurea* (Wang et al., 2013; Wang et al., 2017), using the relevant primers *LaTYDC-F* and *LaTYDC-R* (Table S1) for running PCR, the full length sequence of *LaTYDC1* was identified. The accession number of *LaTYDC1* is MG932082.2.

### Total RNA extraction, cDNA first-strand synthesis and qRT-PCR

100 mg of fresh tissue were fully ground in liquid nitrogen and 1 ml RNAiso was added following the RNA extraction protocol described in the instructions (Takara, Dalian, China). After the extracted RNA was measured using a UV spectrophotometer and 1% (w/v) agarose gel electrophoresis, they were used as templates to create the cDNA transcribed in reverse as previously described by Xu et al. (2016). For the qRT-PCR tests, the DNA sequence of primers of *LaTYDC1* and *LaTIP41* are listed in Table S1. The qRT-PCR program started at 95 °C for 5 min, then took place in a condition of 95 °C for 15 s, 56 °C for 15 s, 72 °C for 20 s, for 40 cycles. The *L. aurea TIP41* gene was applied as a reference gene (Ma et al., 2016, the sequence of TIP41 is shown in Supplemental Information 1).

### miRNA first-strand synthesis and qRT-PCR for microRNA

Reverse transcription was carried out with Mir-X™ miRNA First-Strand Synthesis kit based on a SYBR qRT-PCR user manual for cDNA synthesis (Takara, Dalian, China). Real-time fluorescence quantitative PCR was performed using qTPWER (Analytik Jena, Jena, Germany). The *L. aurea* U6 gene was used for normalization. The qRT-PCR protocol was as follows: 95 °C for 30 s, 40 cycles of 95 °C for 5 s, 56 °C for 15 s, 72 °C for 20 s. In order to verify the specificity of the primers, a melting-curve was also analyzed. Each qPCR was repeated three times.

### Analysis of RNA ligase-mediated-rapid amplification of cDNA ends (RLM-RACE)

Total RNA was extracted and purified from the leaves of *L. aurea* as previously described, followed with further purification with the TRIzol™ Plus RNA Purification Kit (Invitrogen, Carlsbad, CA, USA). The purified RNA was amplified for the RLM-RACE experiment using a First Choice™ RLM-RACE Kit (Invitrogen, Carlsbad, CA, USA), following the method described by Shen et al. (2014). The ligation of 5′ RACE adapter and oligo RNA were performed following the manual of the kit. By using the gene specific primers listed in Table S1 with the 5′ RACE outer or inner primer, nested PCR was performed. The PCR products were agarose gel purified and cloned into pGEM-T vector (Promega, Madison, WI, USA) for sequencing.

### Subcellular localization

The complete open reading frame fragment of *LaTYDC1* was cloned into the expression vector pAN580 and followed by green fluorescent protein (GFP). The construct was transformed into *Arabidopsis* protoplasts as previously described (Zhang et al., 2014). After incubation for 16 h, the expressing cells were observed under an LSM 780 confocal laser scanning microscope (Zeiss, Oberkochen, Germany) with the 488 nm excitation and 505–530 nm band-pass emission filter (Xu et al., 2017).

### Expression and purification of recombinant LaTYDC1

Heterologous expression and protein purification of recombinant LaTYDC1 were carried out as described by Zhang et al. (2011) with some modification. The full length of *LaTYDC1* without the termination codon was cloned into the expression vector pGEX4T-1, followed by GST tag. This recombinan expression plasmid was confirmed by sequencing before being introduced into *E. coli* BL21 (DE3). The transformed bacteria were grown at 37 °C with shaking (180 rpm) in 500 mL of Luria-Bertani medium (Kawalleck et al., 1993) containing ampicillin (50 µg mL^−1^). When the OD600 of the bacteria reached 0.6∼0.8, isopropyl thio-b-D-galactopyranoside (IPTG) at a final concentration of 0.5 mM was added and the incubation conditions were adjusted to 16 °C, 100 rpm. After incubation for 20 h, cells were harvested by centrifugation (4,000 rpm) for 10 min, suspended in 10 ml of 50 mM Tris-HCl (pH 8.0), and then sonicated on ice for 30 min. The homogenate was centrifuged at 12,000 rpm for 20 min at 4 °C, and then the supernatant was passed through a column of high affinity GST purification medium (GenScript, Nanjing, China). The column was washed with 50 mM potassium phosphate buffer (pH 8.0), then the fusion protein was eluted by elution buffer, which contains 50 mM Tris-HCl (pH 8.0) and 0.01 M reduced glutathione.

### Enzyme activity and substrate-specificity analyses

The catalytic activity of the purified GST-LaTYCD1 protein were analyzed followed the method of Lehmann & Pollmann (2009) with some modification. 150 µL mixture containing 15 µg fusion protein, 10 mM pyridoxal-5-phosphate, 15 µL of 20 mM L-tyrosine or L-Dopa and 50 mM Tris-HCl (pH 8.0), and was incubated at 37 °C for 40 min. The reaction was stopped by adding 300 µL of methanol or boiling. Then the mixture of reaction was clarified by passing it through a 0.22 µm pore size nylon membrane filter.

Tyramine and dopamine were analyzed by High Performance Liquid Chromatography (HPLC, Shimadzu LC-20AT, Tokyo, Japan) on a reverse phase column (InertSustain C18, 5 µm, 4.6 mm × 250 mm). To measure tyramine, the solvent composition was: mobile phase A, water with 0.1% phosphoric acid; mobile phase B, 100% acetonitrile. The eluent gradient was: 10% B to 15% B in 25 min, from 15% B to 100% B in 1 min, kept for 10 min, from 100% B to 10% B in 1 min, kept for 15 min, 0.6 mL/min flow rate. The injection volume was 10 µL, and tyramine was detected at 275 nm. To measure dopamine, the solvent composition was: mobile phase A, water with 0.1% formic acid; mobile phase B, 100% acetonitrile. The eluent gradient was: 10% B to 15% B in 15 min, from 15% B to 100% B in 5 min, kept for 5 min, from 100% B to 10% B in 5 min, kept for 10 min, 0.6 mL/min flow rate. The injection volume was 10 µL, and dopamine was detected at 275 nm. Quantification of the target compound was performed using the calibration curves. Varying substrate concentration was used to determine the *K*m and Vmax values (Kang et al., 2007).

### Determination of tyramine in plants by HPLC

Fresh plant materials were weighted and totally ground. 1 g of the ground sample was extracted with 25 mL of methanol in a glass tube, and all of these tubes were placed in an ultrasonic bath for 8 h (Zhou & Su, 2008). The supernatants were clarified by passing through a 0.22 µm pore size NC filter and tyramine was detected by HPLC.

To analyze the tyramine of the extracts by HPLC, the solvent composition was: mobile phase A, water with 0.3% phosphoric acid; mobile phase B, 100% acetonitrile. The eluent gradient was: 5% B for 15 min, then the eluent from 5% B to 100% B in 5 min, kept for 10 min, then from 100% B to 5% B in 5 min, kept for 10 min, 0.8 mL/min flow rate. The injection volume was 10 µL, and tyramine was detected at 275 nm. Quantification of tyramine was performed using the calibration curves.

### Phylogenetic analysis

Phylogenetic tree analysis of LaTYDC1 and TYDCs proteins from different species by using Maximum likelihood method. The phylogenetic tree constructed using MEGA5.5 software comparsion of amino acid sequences of plant EgTYDC1 (*Erythranthe guttata*, XP_012827457.1); NnTYDC2 (*Nelumbo nucifera*, XP_010245171.1); DcTYDC2(*Dendrobium catenatum*, PKU83762.1); QsTYDC1(*Quercus suber*, SXP_023871760.1); AcTYDC (*Aristolochia contorta*, DQ986331.1); AsTYDC2 (*Apostasia shenzhenica*, PKA66030.1); AcTYDC2 (*Ananas comosus*, OAY76026.1); RcTYDC1 (*Rosa chinensis*, XP_024161133.1); JrTYDC1 (*Juglans regiatyrosine*, XP_018819733.1); LaTYDC1(*Lycoris aurea*, MG932082.1); NpTYDC1 (*Narcissus pseudonarcissus*, AUG71932.1); PbTYDC1 (*Pyrus bretschneideri*, XP_009377476.1); NpTYDC2 (*Narcissus pseudonarcissus*, AUG71933.1); RsTYDC (*Rhodiola sachalinensis*, ABF06560.1); OsTYDC (*Oryza sativa*, XP_015644906.1); PcTYDC (*Petroselinum crispum*, AAA33862.1); PaTYDC1(*Prunus avium*, XP_021812417.1); PsTYDC7 (*Papaver somniferum,* AF025434.1); AmTYDC (*Argemone mexicana*, ACJ76782.1); AtTYDC (*Arabidopsis thaliana*, NP_001190862.1); TcTYDC (*Theobroma cacao*, EOX96928.1).

### Statistical analysis

Statistical analysis was performed using SPSS 13.0, and differences were analyzed with one-way ANOVA test or *t*-test. Statistical significance was assumed at *P* < 0.05.

## Results

### MeJA treatment resulted in more tyramine accumulation in *L. aurea* plants

**Figure 2:**
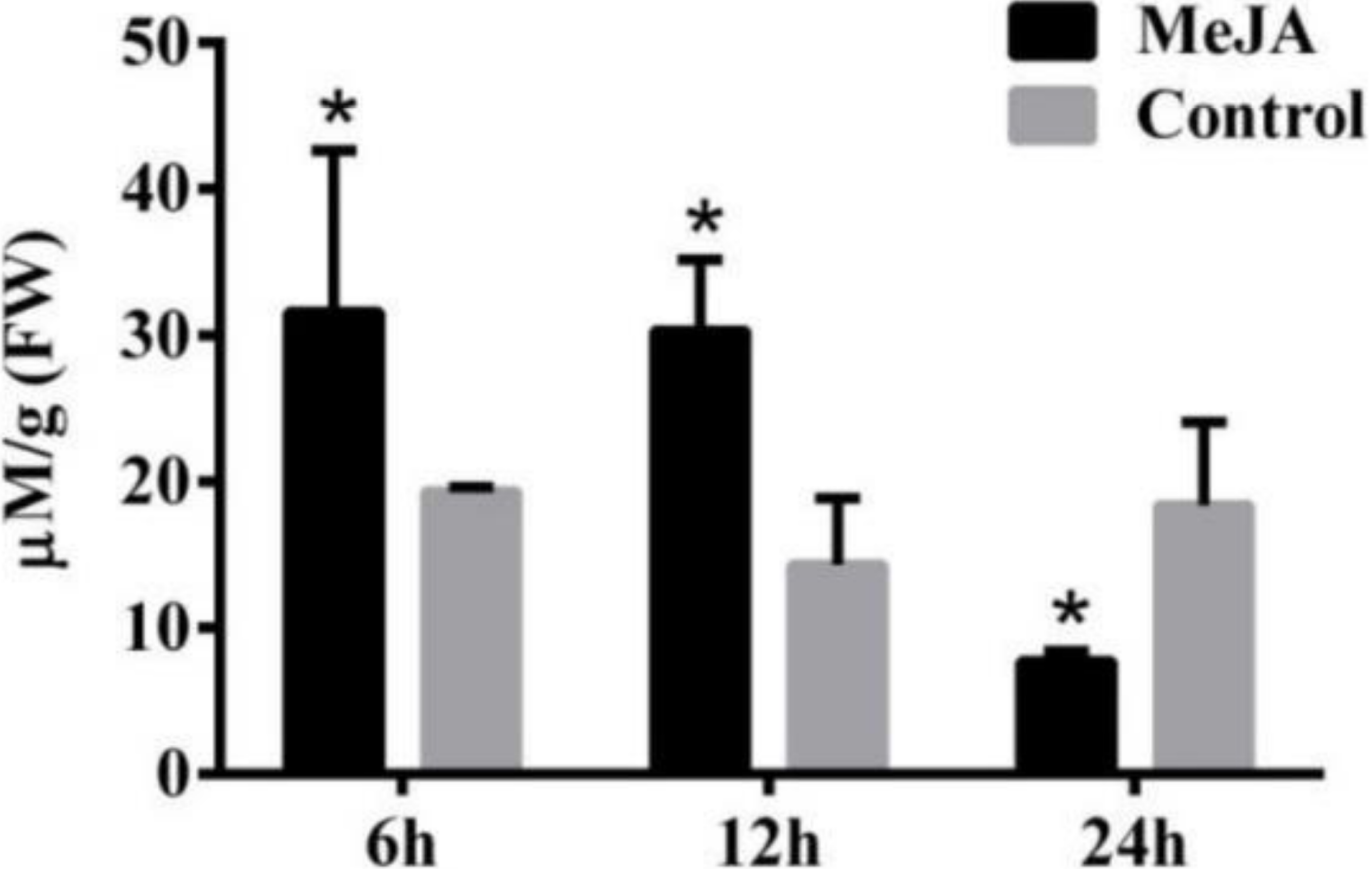
The content of tyramine in root under MeJA treatment. *L. aurea* seedlings showed an increased tyramine under MeJA treatment. The seedlings were treated with 100 µM MeJA for 6, 12 and 24 h (black), Dimethyl sulfoxide (DMSO) was used as vehicle control (gray). Error bars represent SE (*n* = 3). Statistical difference were estimated using independent *t*-test compared with the respective controls. Differences are significant with *p* value <0.05(*) (*p*-value =0.027 at 6 h, *p*-value =0.014 at 12 h and *p*-value =0.039 at 24 h, degree of freedom = 3).

Secondary metabolite accumulation is tightly controlled by Jasmonate (JA) signaling (Wasternack, 2007), and our previous results show that MeJA induces the accumulation of galanthamine in *Lycoris chinensis* Traub (Mu et al., 2009). In this study, 100 µM MeJA were used to treat the *L. aurea* seedlings for 24 h, then the contents of tyramine in the roots were measured by HPLC. As the results show in Fig. 2, the seedlings accumulated 31.59 µmol g^−1^ tyramine in the roots after 6 h of MeJA treatment, when the control sample accumulate 19.32 µmol g^−1^ tyramine. After 12 h of MeJA treatment, tyramine content of the treated sample was 30.31 µmol g^−1^ , almost two-folds the amount in the control samples. After 24 h, the content of tyramine in experimental seedlings decreased to 7.7 µmol g^−1^, almost one half of the control samples.

### Characterization of *LaTYDC1*

**Figure 3:**
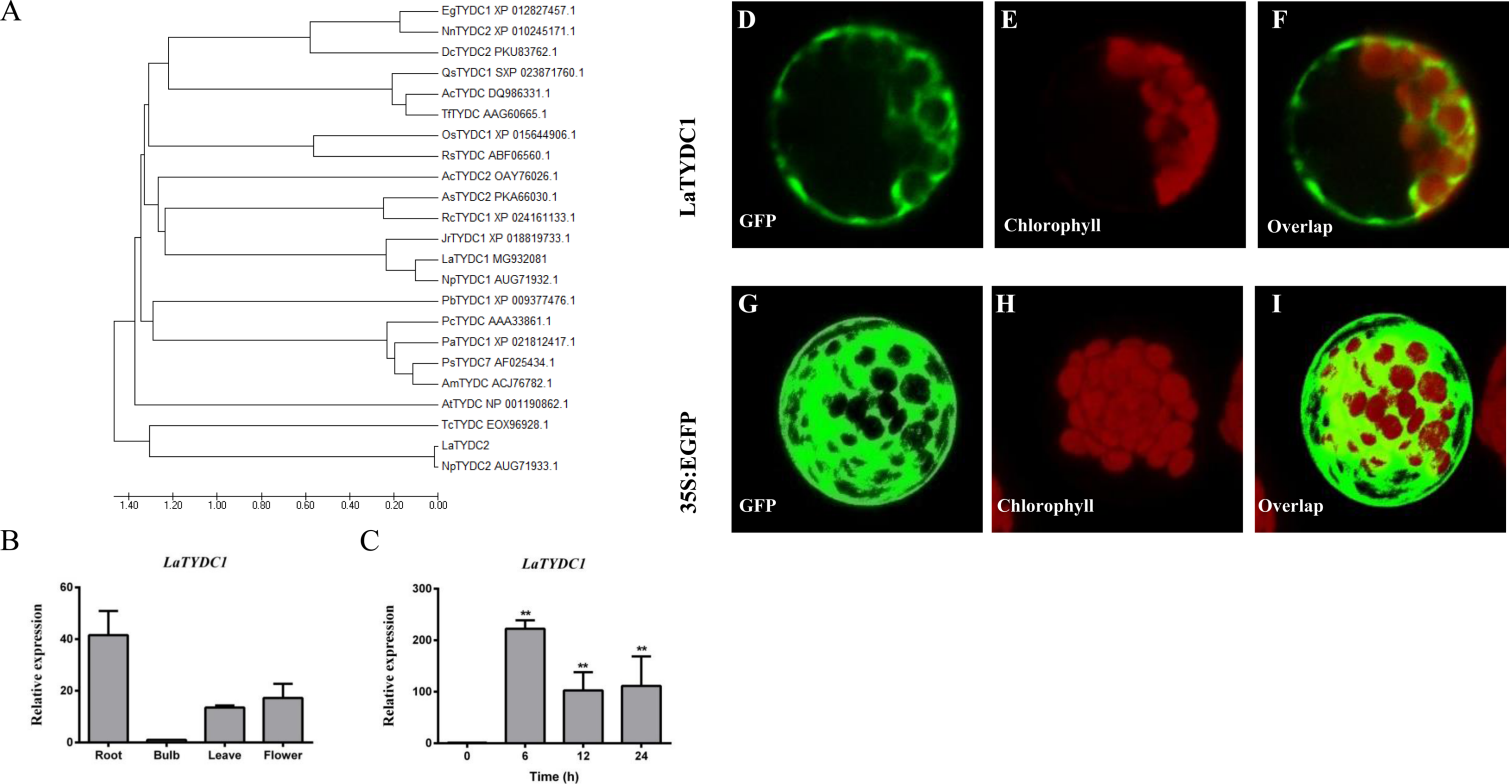
Characterization of LaTYDC1 protein. (A) The phylogenetic tree constructed using MEGA 5.5 software. (B), The expression pattern of LaTYDC1 analyzed by qRT-PCR. Error bars represent SE (*n* = 3). (C), Time course expression analysis of LaTYDC1 in response to MeJA (100 µM) treatment. The expression in plants without MeJA was used as a reference of basal expression level. Error bars represent SE (*n* = 3). Statistical difference was estimated using Least Significant Difference test compared with the 0 h controls. Differences are significant with *p* value <0.01(**) (*p*-value =0.000 at 6 h, *p*-value =0.007 at 12 h and *p*-value =0.005 at 24 h, degree of freedom = 8). (D–J), Subcellular localization of LaTYDC1. Arabidopsis mesophyll protoplasts were transformed with the combined constraction indicated: (D), a protoplast expressed LaTYDC1-GFP (green), (E), the chlorophyll of this protoplast (red). (F), merged images of (D) and (E). (G) to (I) a protoplasm expressed GFP empty vector. Bar = 10 µm.

*LaTYDC1* covers an open reading frame representing 511 amino acids with a predicted molecular weight of 56.7 kDa. Phylogenetic analysis of LaTYDC along with TYDCs form other species was carried out using maximum likelihood method. We conferred this gene of interest as *LaTYDC1*, which is placed near NpTYDC1 and another TYDC from *L. aurea,* and shares a high similarity with NpTYDC2 (Fig. 3A). In addition, the sequence alignment of these TYDCs indicated that LaTYDC1obviously shared the conserved domain with other TYDC proteins, and the deduced amino acid sequence of LaTYDC1 showed 47–89.67% shared identity with those from other plant origins (Fig. S1). *LaTYDC1* expression in root, leaf and flower were 40, 10, and 15-fold greater, respectively, than that in bulb (Fig. 3B). At the beginning, six hours after 100 µM MeJA treatment, the accumulation of *LaTYDC1* significantly increased almost 200 folds when compared with the vehicle control samples, and sharply decreased when compared with the expression level of samples 6 h after MeJA treatment. The similar results were observed after 24 h treatment (Fig. 3C).

To further explore the function of LaTYDC1, we transiently expressed the LaTYDC1-GFP (green fluorescent protein) fusion protein in the Arabidopsis protoplast. As the green fluorescence signal was observed at the cytoplasm, it showed that LaTYDC1 localizes at the cytoplasm (Fig. 3D).

### Enzymatic characterization of LaTYDC1

**Figure 4:**
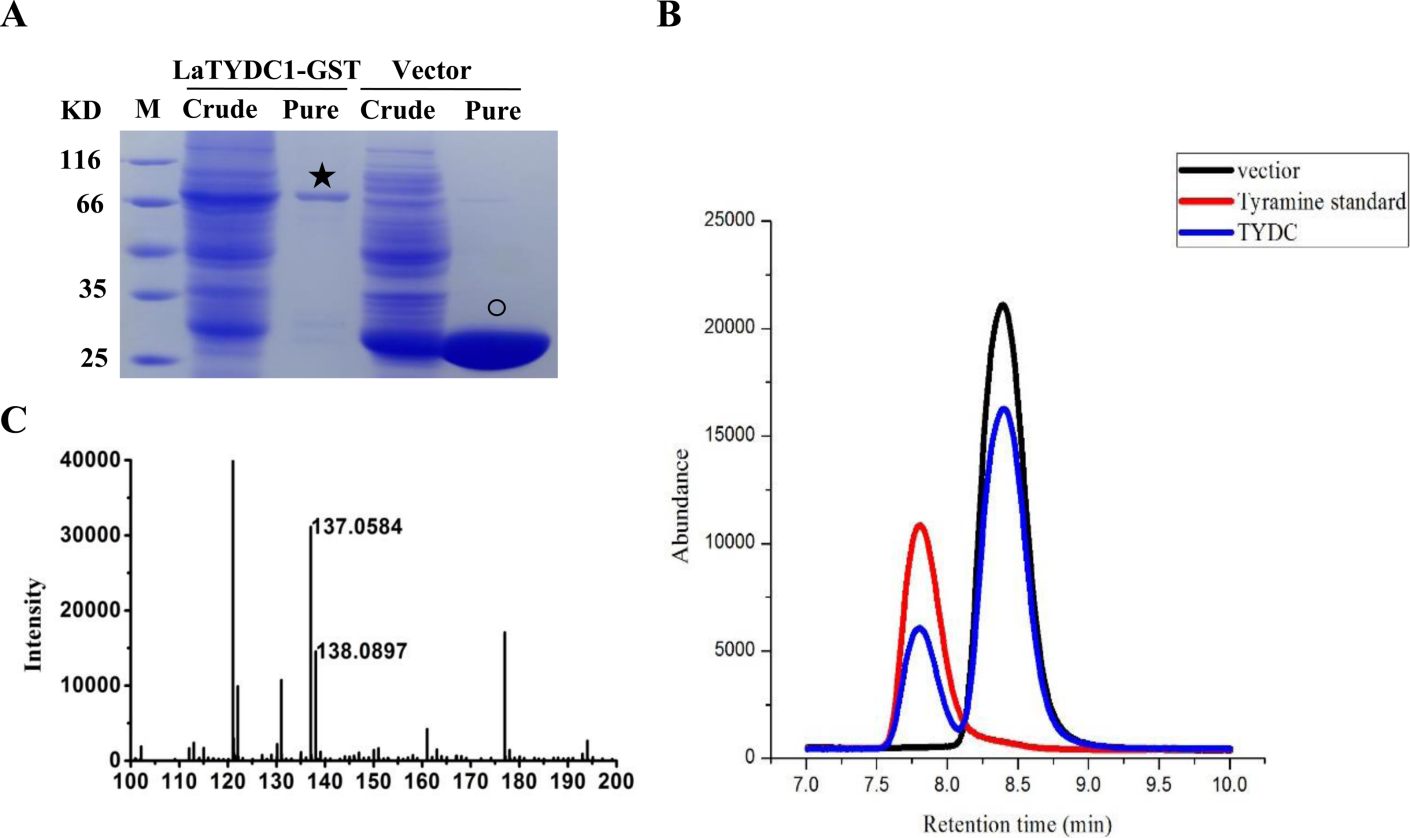
Enzyme assay of LaTYDC1. (A), Analysis the proteins with denaturing SDS-PAGE. The lanes showed the crude extracted or purified proteins. The star indicates the LaTYDC1-GST protein. The opened circle indicates the GST protein. (B), Enzyme assays by using HPLC. Red line: tyramine standard; Black line: Enzyme assay with *E. coli* vector only purified protein, the peak stands for the substrate tyrosine; Blue line: Enzyme assays with LaTYDC1 protein. (C), MS analysis of the product tyramine, 137 *m/z*, indicate the tyramine, and 138 *m/z* present for the tyramine with one hydrogen.

To examine the enzymatic function of LaTYDC1, we expressed the LaTYDC1 protein fused with a 26.8 kDa GST tag in *E. coli* BL21. As shown in Fig. 4, after purification using a high affinity GST purification column, the LaTYDC1-GST fusion protein was checked by SDS-PAGE, and the fusion protein showed a predictable molecular weight (MW) of 83.5 kDa (Fig. 4A).

Assuming that LaTYDC1 has reductase activity, we were interested in testing whether the fusion protein could catalyze tyrosine into tyramine conversion. HPLC analysis was then carried out with the samples for the enzyme assay, in which the purified LaTYDC1 fusion protein uses tyrosine as substrate. The result shown in Fig. 4B demonstrated that the fusion LaTYDC1 was able to specifically catalyze the synthesis of tyramine, since the product with an identical peak in regards to retention time was the same as the corresponding tyramine standard (Fig. 4B). In addition, this product was determined by GC-MS analysis, and it was confirmed that the product is tyramine (Fig. 4C). Moreover, the optimal temperature for LaTYDC1 enzyme activity is 50 °C; when the temperature is below 30 °C or above 55 °C, enzyme activity rapidly declines (Fig. S2A). The optimum pH of LaTYDC1 is 8.5; when the pH value is above 9, enzyme activity sharply declines (Fig. S2B). Furthermore, the substrate affinity was determined by calculating the *K*_m_ we used tyrosine and 3,4-dihydroxy-L-phenylalanine (Dopa) as the substrate for LaTYDC1. The *K*_m_ for tyrosine is 602.34 ± 38 µM, and the *K*_m_ for dopa is 966.35 ± 79 µM. The Vmax is 3.19 ± 0.51 (nkat mg^−1^) and 1.24 ± 0.095 (nkat mg^−1^), respectively (Table 1).

**Table 1:** Substrate specificity tests for LaTYDC1. The substrate affinity was determined by calculating the *K*_m_, tyrosine and 3,4-dihydroxy-L-phenylalanine (Dopa) were used as the substrate for LaTYDC1.

Substrate	*K*_m_ (µM)	Vmax (nkat mg^−1^)
Tyramine	602.34 ± 0.12	3.19 ± 0.51
Dopa	966.35 ± 79	1.24 ± 0.09

### Identification of *LaTYDC1*-ralated miRNA in *L. aurea*

**Figure 5:**
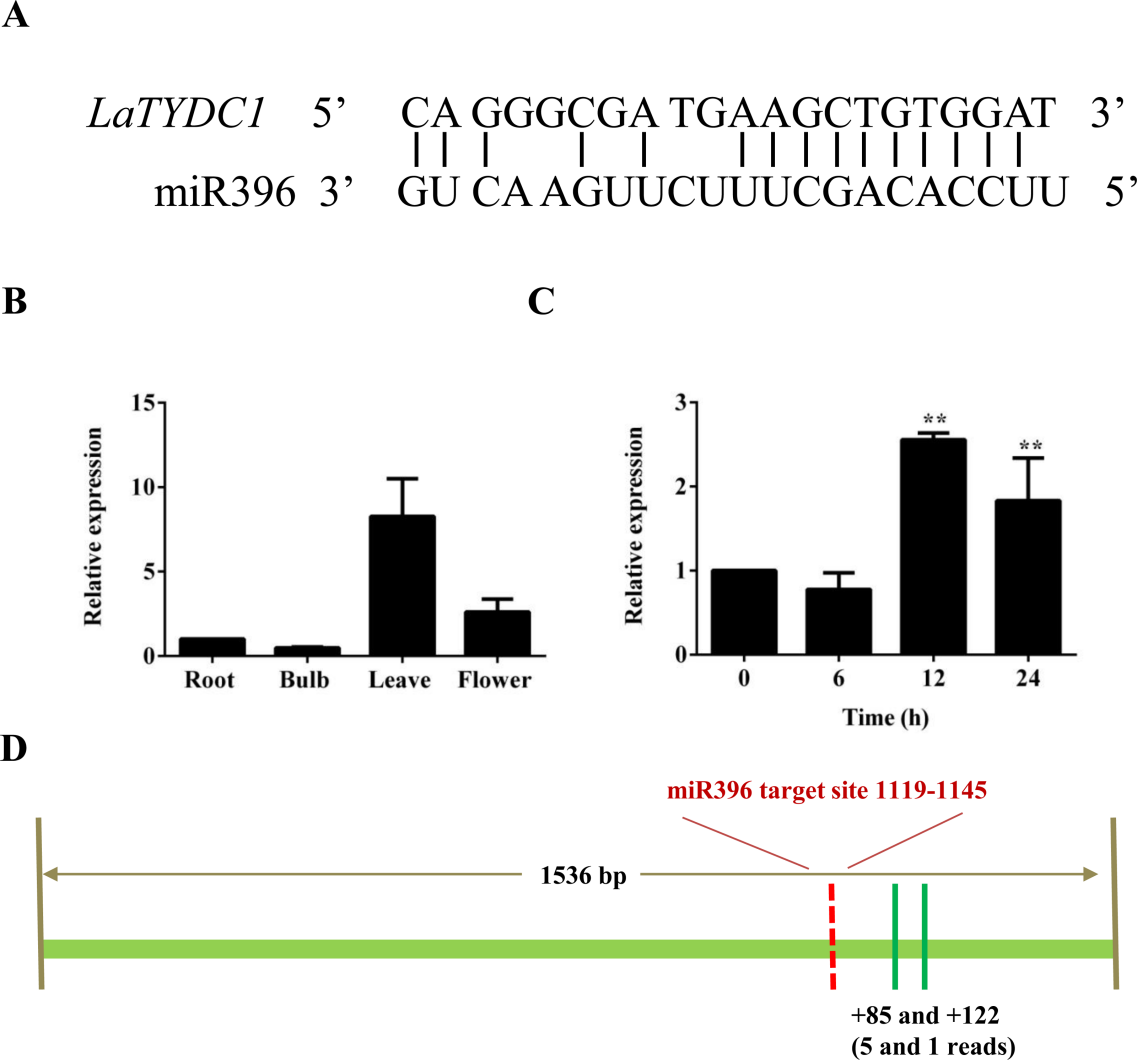
*LaTYDC1* is the proposed target of miR396. (A) The aligments of miR396 with the *LaTYDC1* target sequence. (B) The expression pattern of miR396 analyzed by qRT-PCR. Error bars represent SE (*n* = 3). (C) Time course expression analysis of miR396 in response to MeJA (100 µM) treatment. The expression in plants without MeJA was used as a reference of basal expression level. Error bars represent SE (*n* = 3). Statistical difference was estimated using the Least Significant Difference test compared with the 0 h control. Differences are significant with *p* value <0.01(**) (*p*-value =0.000 at 12 h and *p*-value =0.006 at 24 h, degree of freedom = 8). (D) The validation of miR396-mediated cleavage *of LaTYDC1* by RLM-RACE. The targeting site was indicated with red line and the mapped targeting site was indicated with green lines. Distances relative to the predicted cleavage site and the number of each clone were shown at the bottom.

The sudden change of *LaTYDC1* expression under MeJA attracted our attention. MiRNAs play post-transcriptional regulatory roles in plants, and are involved in the regulation of secondary metabolite biosynthesis in many plants (Zhao et al., 2012). To identify the genes and miRNA that participate in the JA signal transduction pathway and are involved in galanthamine biosynthesis, we searched the response miRNA sequencing database of *L. aurea* (Xu et al., 2016). We identified that miR396 is responsive to MeJA treatment, and the predicted target is *LaTYDC1*. The cleavage site was confirmed by degradome data and is shown in Fig. 5A. qRT-PCR were used to determine the expression level of miR396 in different tissues. The results showed that the expression of miR396 was the lowest in roots, and was mainly expressed in leaves. The expression of miR396 in root, leaf and flower were respectively 2, 17, and 4-fold greater than that in the bulb (Fig. 5B). This co-expression analysis also showed that miR396 was negatively correlated with the target *LaTYDC1*. As miRNA paired with *LaTYDC1* mRNA to direct transcriptional suppression, we did qRT-PCR to determine miR396 expression under MeJA treatment, and the expression of miR396 was repressed about one half of the vehicle sample. Twelve hours after MeJA treatment, the expression of miR396 increased ∼3 folds (Fig. 5C). Combined with the repressed expression of *LaTYDC1*, the reversely correlated expression relationship suggests that miR396 may target *LaTYDC1.* RLM-RACE experiments were carried out to confirm the cleavage, and the cleavage sites were mapped in flanking sequence of the complementary region (Fig. 5D), the DNA sequences in the RLM-RACE experiments were listed in Supplemental Information 2).

## Discussion

It is known that the secondary metabolites of some medical plants are beneficial for human health. Amaryllidaceae alkaloids that come from plants in the Amaryllidaceae family have anti-cancer, anti-virus and acetylcholine esterase inhibitor properties (Bores et al., 1996). These secondary metabolites have been used clinically, but their product yield has been limited since these alkaloids are extracted from plant tissues. The biosynthetic pathway of the Amaryllidaeae alkaloids has been partly investigated (Kilgore et al., 2014; Kilgore et al., 2016). So far, there are two key enzymes that have been found, but the majority of its mechanism has remained unexplored.

TYDC enzymes take part in primary and secondary plant metabolism. In fact, TYDC catalyzes the initial reaction of many secondary metabolites, such as salidroside in *Rhodiolasa chalinensis* (Zhang et al., 2011), verbascoside in *Syringa vulgaris* (Ellis, 1983) and hordenine in *Hordeum vulgare* (Leete & Marion, 2011). In *Lycoris* plants, the aromatic amino acid tyrosine is converted to tyramine by TYDC and provides the basic bone for Amaryllidaceae alkaloids including galanthamine (Kilgore et al., 2014). Tyramine and many secondary metabolites play important anti-stress roles, and the accumulation of them could help the plant get over stress (Lehmann & Pollmann, 2009; Kabera et al., 2014). JAs are the key signals in stress response and trigger secondary metabolite production (De Geyter et al., 2012). Under JA signaling, the increase of galanthamine is significant (Colque et al., 2004; Mu et al., 2009; Ptak et al., 2010; Jiang et al., 2013). Meanwhile the content of tyramine in *L. aurea* seedlings was almost two fold when compared with the control (Fig. 2), and the expression of LaTYDC1 was activated (Fig. 3C). In addition, the expression pattern of *LaTYDC1* was in line with the accumulation pattern of galanthamine (Fig. 3B; Fig. S3). All of these results indicated that under MeJA treatment, the accumulation of galanthamine is associated with the increase of tyramine content.

Several TYDC genes from different plants have been isolated and characterized (Kawalleck et al., 1993; Facchini & De Luca, 1994; Lehmann & Pollmann, 2009; Zhang et al., 2011). TYDCs use tyrosine and dopa as substrates, but are not able to catalyze L-phenylalanine (Compant, Clément & Sessitsch, 2010; Facchini & De Luca, 1994; Facchini, Huber-Allanach & Tari, 2000). To determine the enzymatic function and substrate specificity of LaTYDC1, we chose tyrosine and dopa as substrates. The results showed that LaTYDC1 could convert tyrosine to tyramine (Fig. 4B), and have a higher substrate specificity for tyrosine over dopa (Table 1).

In previous reports, when the gene *RsTYDC* in *Rhodiolasa chalinensis* was overexpressed, the content of tyramine and salidroside in transgenic seedlings was higher than in the wild type. This showed that final product yield is associated with the expression level of the initial enzyme in the biosynthetic pathway (Zhang et al., 2011). In this study, under MeJA treatment, the content of tyramine was just double fold when compared with the untreated seedlings (Fig. 2), but the transcription of *LaTYDC1* enormously increased (Fig. 3C). The enzyme activity was not correctly in line with the transcriptional level of *LaTYDC1*, most likely due to post-transcriptional modification. The stability of mRNA (Parker & Song, 2004; Wilusz, Wormington & Peltz, 2001), the half-life of mRNA, the regulation of the translation (Preiss & Hentze, 2003), and so on, create a varied and complex mechanism of post-transcriptional regulation that controls gene expression (Mata & Al, 2005; Roundtree & He, 2016).

MiRNAs negatively modulate the expression of targeted genes through direct cleavage (Li et al., 2014). In our results, miR396 was identified from the *L. aurea* miRNA profile, which is responsive to MeJA treatment. By analyzing the degradome sequencing data, miR396 targets *LaTYDC1* (Fig. 5A). At the beginning of the MeJA treatment, the expression of *LaTYDC1* greatly increased (Fig. 3C); meanwhile miR396 was still at the lower level as the control (Fig. 5C). However, with the treatment alone, the expression of miR396 was elevated to a higher level, and the expression of *LaTYDC1* was decreased to only one half of the 6-h treatment samples. Based on our previous miRNA sequencing work (Xu et al., 2016), along with the expressional relationship between miR396, *LaTYDC1*, and the degradome sequencing data together, it is reflected that miR396 negatively regulates *LaTYDC1*.

In Arabidopsis, seven of the GRFs contain the cleavage sites recognized by miR396. These miR396-GRFs target interaction, control the proliferation of cells in leaves, pistil development and the switch between stem cell and transit-amplifying cells in roots (Debernardi et al., 2014; Liang et al., 2014; Rodriguez et al., 2015). Also, miR396 regulate other transcription factors as bHLH74, which is necessary for normal development of Arabidopsis (Debernardi et al., 2012). In this work, we reported the regulation of an additional target of miR396, the enzyme LaTYDC1. According to the degradome data, in *LaTYDC1* gene there is one binding site of miR396, and the cleavage site is shown in Fig. 5A. However, the RLM-RACE experiment failed to confirm this cleavage site, although we repeated the experiment several times with different primers. After sequencing the DNA fragments, the cleavage site was not positioned in the predicted target region. Two out of five reads were mapped at the 85th, and the other three reads were at the 122nd nucleotide downstream of the predicted cleavage sites (Fig. 5D). Although we cannot explain the possibility that we failed to get the right cleavage products, the phenomenon that RLM-RACE mapped the cleavage sites beyond the predicted target region was also observed for other miRNAs (Li et al., 2015; Shen et al., 2014). It is likely that these fragments that resulted from miRNA cleavage are very susceptible to degradation induced by RNA decay proteins (Li et al., 2015).

## Conclusion

This study identified LaTYDC1, which is involved in the biosynthesis pathway of galanthamine. It catalyzes tyrosine to tyramine conversion to provide the base bone of Amaryllidaceae alkaloids. Additionally, we found that miR396 may negatively regulate the expression of *LaTYDC1* to participate in galanthamine biosynthesis. In the future, this finding may also have implications for genetic strategy in increasing the yield of galanthamine.

##  Supplemental Information

10.7717/peerj.6729/supp-1 Table S1 Primer summarization

10.7717/peerj.6729/supp-2 Figure S1 Sequence alignment of the deduced TYDCs protein with known homologs The comparison was conducted by DNAMAN (version 6.0). Amino acid residues conserved in all four sequences are shaded in black, and those conserved sequences are shaded in light gray.

10.7717/peerj.6729/supp-3 Figure S2 The optimum pH and temperature of LaTYDC1

10.7717/peerj.6729/supp-4 Figure S3 The content of galanthmine in different tissues of *L. aurea*

10.7717/peerj.6729/supp-5 Supplemental Information 1 Supplemental information S1

10.7717/peerj.6729/supp-6 Supplemental Information 2 DNA sequences in the RLM-RACE experiments

10.7717/peerj.6729/supp-7 Data S1 Dataset 1 Raw data which were selected for drawing the figures.

## Additional Information and Declarations

### DNA Deposition

The following information was supplied regarding the deposition of DNA sequences: The LaTYDC1 gene sequences described here are accessible via GenBank accession numbers MG932082.1.

### Data Availability

The following information was supplied regarding data availability: The DNA sequence of LaTYDC1, LaTYDC2 and TIP4 is available in Supplemental Information 1. The DNA sequences in the RLM-RACE experiments is available in Supplemental Information 2. These DNA sequences were used to determine the cleavage site targeted by miR396 in LaTYDC1 gene. The raw data are available in Dataset S1. These data provide the data source of all the figures listed in the manuscript, including the supplemental figures.
