# Supplementary material for: Cloning and characterization of a tyrosine decarboxylase involved in the biosynthesis of galanthamine in Lycoris aurea
Source: PeerJ. 2019 Apr 16;7:e6729. doi: 10.7717/peerj.6729 (PMC6474336; doi:10.7717/peerj.6729)
Supplement: Table S1 [file peerj-07-6729-s001.docx]

**Table S1 Primer summarization**

| **Primer** | **Sequence** |
| --- | --- |
| *LaTYDC*-F | CCGAGCACAGGCCCGGGAGCTGG |
| *LaTYDC*-R | TTGTAGCCGTTTGTGTTCCCCCAGCAA |
| *LaTYDC*-pAN580-F | GTCTTAAGTCCGGAGCTAGCTCTAGAATGGGCAGCCTTGGCTCTGATAATA |
| *LaTYDC*-pAN580-R | TCCTCGCCCTTGCTCACCATGGATCCTTGTAGCCGTTTGTGTTCCCCCAGCAA |
| *LaTYDC*-pGEX4t-1F | ATCGGATCTGGTTCCGCGTGGATCCATGGGCAGCCTTGGCTCTGATAATA |
| *LaTYDC*-pGEX4t-1R | ATCGTCAGTCAGTCACGATGCGGCCGCTCATTGTAGCCGTTTGTGTTCCCCCAGCAA |
| qRT-*LaTYDC*-F | CTGCGAGAGACAGGGTACTGAATA |
| qRT-*LaTYDC*-R | CACCTTTTGTAGCTCCTCAGGATT |
| *TIP41*-F | GCAACCATCCAAAGTTTAACTGCT |
| *TIP41*-R | AATGTGCAAGCAGGGCTAGTAA |
| miR396 | TTCCACAGCTTTCTTGAACTG |
| mRQ 3’ Primer | TAKARA Primers |
| U6 snRNA | TAKARA Primers |
