# Supplementary figures and images for: Cloning and characterization of a tyrosine decarboxylase involved in the biosynthesis of galanthamine in Lycoris aurea
Source: PeerJ. 2019 Apr 16;7:e6729. doi: 10.7717/peerj.6729 (PMC6474336; doi:10.7717/peerj.6729)

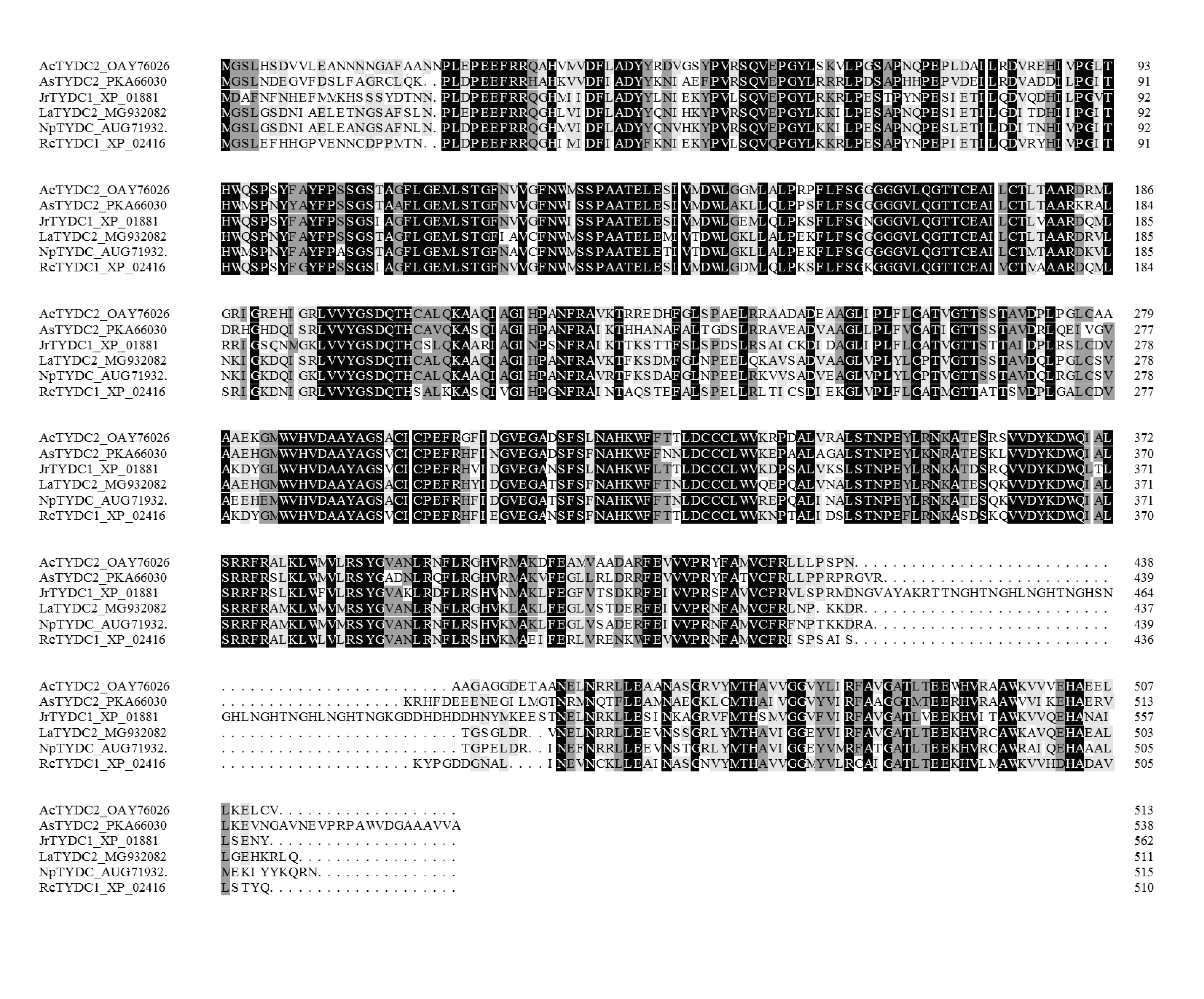

Supplement: Figure S1 — The comparison was conducted by DNAMAN (version 6.0). Amino acid residues conserved in all four sequences are shaded in black, and those conserved sequences are shaded in light gray. [file peerj-07-6729-s002.jpg]

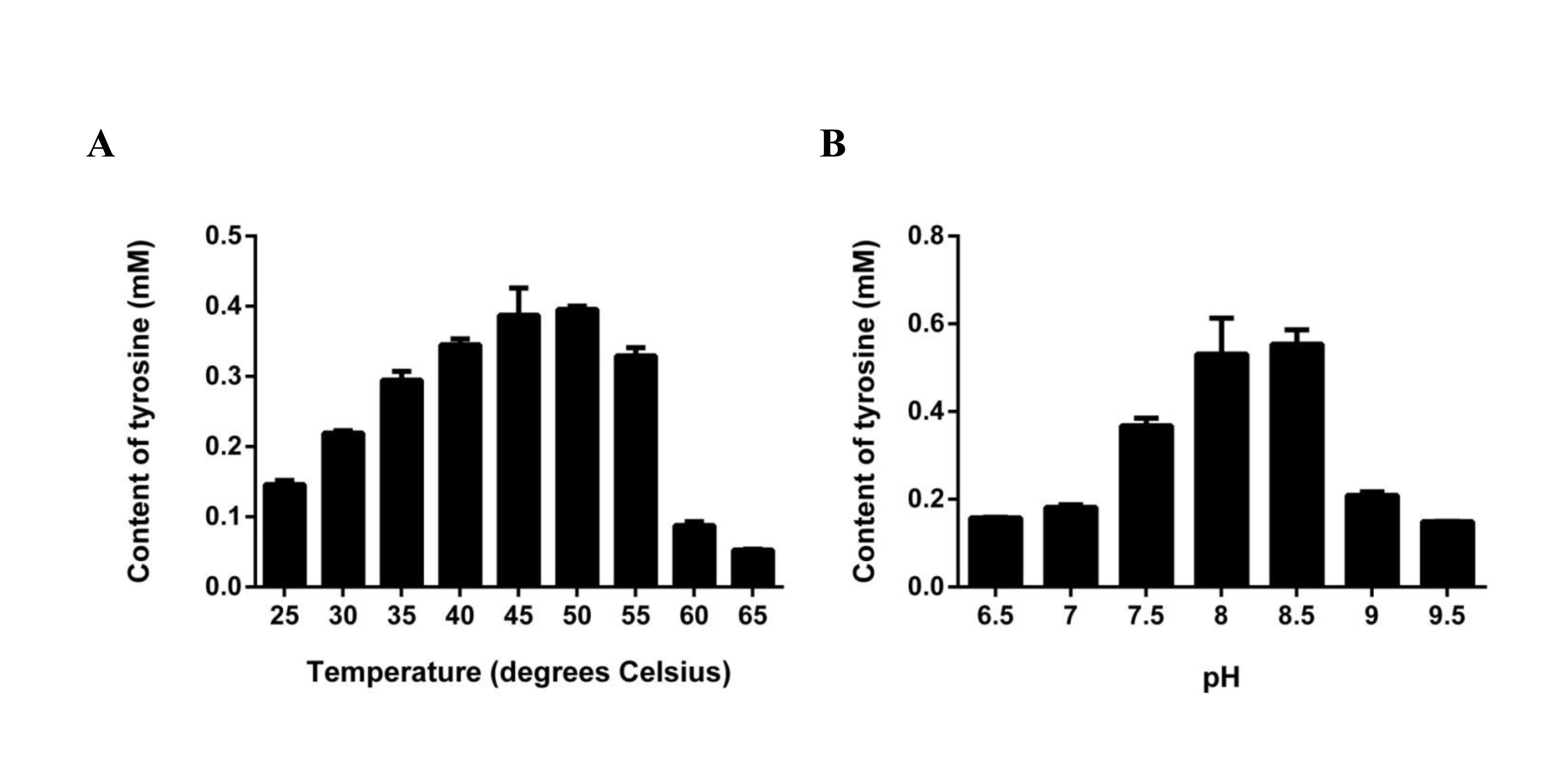

Supplement: Figure S2 [file peerj-07-6729-s003.jpg]

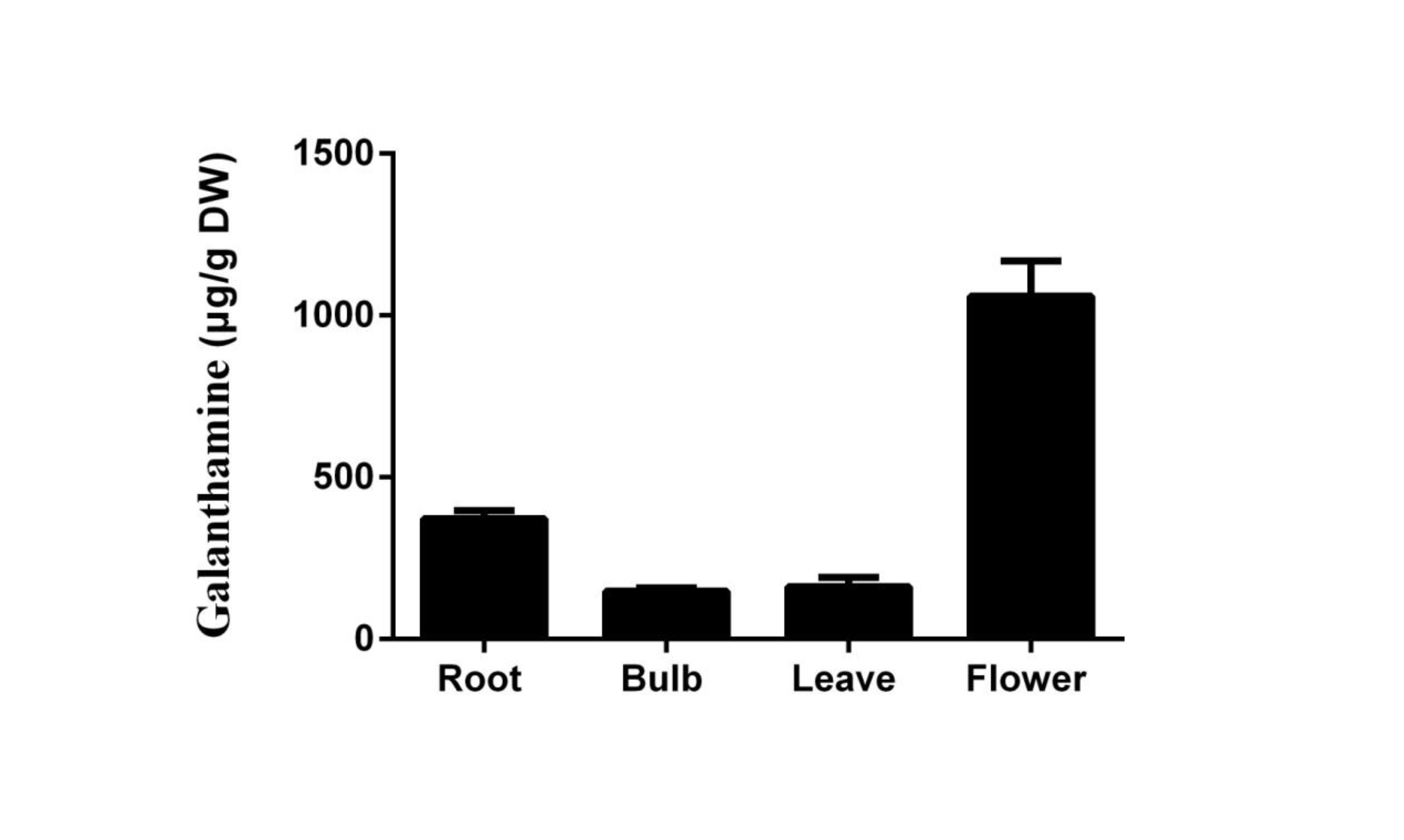

Supplement: Figure S3 [file peerj-07-6729-s004.jpg]
