# Supplementary material for: Cloning and characterization of a tyrosine decarboxylase involved in the biosynthesis of galanthamine in Lycoris aurea
Source: PeerJ. 2019 Apr 16;7:e6729. doi: 10.7717/peerj.6729 (PMC6474336; doi:10.7717/peerj.6729)
Supplement: Supplemental Information 1 [file peerj-07-6729-s005.docx]

LaTYDC1 (MG932082.1)

ATGGGCAGCCTTGGCTCTGATAATATTGCTGAGCTAGAAACAAATGGCTCTGCATTCTCTCTGAATCCCCTCGAGCCCGAGGAATTCCGTCGACAAGGCCACTTGGTCATCGACTTCCTCGCTGATTACTACCAAAACATCCACAAGTACCCCGTCCGTAGCCAGGTCGAACCAGGCTACTTAAAAAAGATCCTGCCCGAATCCGCTCCGAACCAACCCGAATCCATCGAAACCATTCTCGGCGACATCACCGACCACATCATCCCCGGCATCACACACTGGCAGAGCCCTAATTATTTCGCCTACTTCCCTTCTAGTGGGAGCACGGCGGGCTTTCTCGGCGAGATGCTGAGCACGGGATTTATCGCCGTGTGCTTTAACTGGATGTCATCGCCTGCGGCTACCGAGTTGGAGATGATCGTGACGGATTGGCTTGGGAAGCTGTTGGCATTGCCCGAAAAGTTCTTGTTCTCCGGCGGTGGCGGTGGAGTCTTGCAAGGTACTACTTGCGAGGCGATTCTGTGTACGTTAACTGCTGCGAGAGACAGGGTACTGAATAAGATTGGAAAAGATCAGATCA

GTAGGCTCGTCGTCTACGGATCGGATCAGACCCACTGTGCCCTGCAGAAGGCTGCTCAGA

TTGCCGGCATTCATCCCGCAAACTTCCGCGCCGTCAAAACTTTCAAATCTGACATGTTCG

GCCTCAATCCCGAGGAGCTACAAAAGGCGGTGTCAGCCGACGTGGCGGCCGGGCTTGTCC

CGCTGTATCTTTGTCCGACGGTGGGGACCACGTCGTCGACCGCGGTGGACCAGCTCCCGG

GCCTGTGCTCGGTGGCTGCGGAGCACGGGATGTGGGTCCACGTAGACGCAGCGTACGCGG

GCAGCGCGTGCATCTGCCCAGAGTTCCGGCACTACATCGACGGGGTGGAGGGGGCGACAT

CCTTCAGCTTCAACGCGCACAAGTGGTTCTTTACAAACCTCGACTGCTGCTGCCTCTGGG

TCCAAGAACCGCAGGCATTGGTCAACGCCCTGTCGACCAACCCAGAGTACCTACGGAACA

AGGCGACCGAGTCCCAGAAGGTGGTTGACTACAAAGACTGGCAGATTGCCCTCAGCCGTC

GGTTCAGGGCGATGAAGCTGTGGATGGTGATGCGGAGCTACGGCGTGGCGAATCTACGAA

ACTTCTTACGGGGTCATGTTAAGTTGGCCAAGCTATTCGAAGGGTTGGTGTCGACGGATG

AAAGGTTCGAGATCGTAGTTCCGAGGAACTTCGCCATGGTCTGCTTCCGGCTCAACCCCA

AAAAGGACCGAACAGGGTCGGGATTGGACCGGGTCAACGAGCTGAACCGGAGGTTGCTGG

AGGAGGTGAACTCGTCGGGGAGATTGTACATGACCCATGCAGTGATCGGAGGCGAGTACG

TGATCAGATTCGCAGTGGGGGCCACGCTCACCGAGGAGAAGCACGTGCGATGCGCGTGGA

AAGCCGTGCAGGAGCATGCAGAAGCTTTGCTGGGGGAACACAAACGGCTACAA

LaTYDC2

CTCTCTCTCTCTCTCTCTCTCTTCTCTAGGGTTTTTCTTCATCAAAATTC

ATCATTCTAATGGAGAAATCTTTGAAGCCGATGGATGCAGAACAATTGAG

AGAGAATGCTCACAAGATGGTTGATTTCATCGCTGATTACTACAAGAGCA

TAGAATCTTTTCCTGTTCTCAGCCAAGTTAAGCCGGGGTATCTAAGGGAT

CTTCTTCCTGATTCAGCCCCTGACCATCCTGAAAGTCTTGAAGATGTTCT

AGAAGATATTCGCCAGAAGATTGTGCCAGGCGTAACCCATTGGCAAAGCC

CCAATTATTTTGCCTATTATCCGTCTAACAGTAGCGTAGCTGGATTCCTT

GGAGAAATGATCAGTGCTGGTTTTAATATTGTGGGTTTCAATTGGATGGC

CTCTCCCGCTGCCACAGAGCTCGAGGTTATTGTTTTGGATTGGCTTGCTA

AAATGCTGAACTTACCAAACCAATTCCTTTCTTCTGGGCAGGGTGGTGGA

GTAATTCAAGGCACAGCTAGTGAAGCTAACCTTGTTGTATTGTTGGCTGC

TCGTGATAAATTCTTGAAGAGATTTGGGAAAAGATCACTTGAAAAGCTTG

TGGTTTATGCTTCAGATCAAACACATGCAGCTATGAAAAAGGCATGCCAG

ATTGCAGGAATCTATCCTGAAAACTTTAGGGTGCTCAATGCTGACCACAG

TTCAAACTATGCTCTTGTTCCGGAAGCACTCTCAGATGCAATTTCTAATG

ATTTGTCAGCAGGCTTAATACCATTCTTCCTCTGTGCTACAGTTGGAACT

ACATCTTCTGCTGCTGTTGATCCCTTATCAGAGTTGGGGAAGATTTCCAA

GGTTAACGAGATGTGGTTCCATGTCGATGCTGCATATGCTGGCAGTGCTT

GTATTTGCCCAGAATACCGACATTATATAGATGGTATAGAAGAAGCAGCT

TCCTTTAATATGAATGCGCACAAATGGTTTCTTACAAACTTTGACTGCTC

CGTGCTCTGGGTTAAGGATCGAAGTGCTTTAATTCAGTCCTTATCCACAT

ATCCTGAGTATCTAAAAAATAAGGCCTCACAGGAAAATAGAGTTGTGGAT

TTCAAAGATTGGCAAATTCCACTTGGACGCCGTTTCAGATCGTTAAAGCT

ATGGATGGTTCTGAGGTTATATGGTTTAAAAAACCTACAAAGTTACATTC

GTAATCACATAAAGTTGGCTGGGCAATTTGAACAACTTGTCTGTTCTGAT

TCTAGGTTTGAGGTTGTCGCCCCTCGAACTTTTTCTCTTGTTTGCTTCCG

TCTTCTCCCTCCACCCAATCATCAGGATGACGGCTATAAGTTAAATCACA

GCTTACTTGATGCAGTCAATTCTAGTGGGAAAATTTTTGTATCACATACT

GTCTTGTCTGGCAAGTATGTCATTCGATTCGCAGTGGGAGCACCGTTGAC

AGAAGAAGCACATATTAAGCAAGCCTGGAAGGTTTTGCAGGACCAGGCAA

CTATTCTTTTAGCAGGGTCTGATGGCAGTGATTTTGGCACTCGCAACGGA

GACATCATTTGATGGATAACATGCAATACGAAAATTGAAAATTTCCTTAA

AGCTTTATTTGTATAATTGCTGATTGGCTTTGTATTCAAGCAAACTATGT

CATGAAAACCATTGTTTCTTGTTTCTAGCTCCTGTTCTATCGAAGTACGG

CTTTGAATGTGGAATAACATGCATTTGCAACTTTTTTTTGACAAAAAG

TIP4

GTTTTTTCTTAACAGCCCCCAATTTGCCCCAATAATATATCTGTTACCGTAGAGAAAATT

CAAAGAACACTCTCGATCAATGGCGGAATGCGAAGTTGGGAGGGAGATCGATCTAAAAGC

CGCTGGCGCCGTGGTTCTTCCTGATGGACGCCGAGGATTGCGTCTCAAGGGTTGGCAGAT

CGAATCCTTCAATCGCCCCATCCTCGCCTCTCTCGCCTTCCAAGAGTGGGAAGAAAAGCT

TGGAACATCACATTTACCTGAGATGGTTTTTGGAGAGAGCTCTCTGAGTCTTTTACATGT

GGACACTGGTGTTAAATTACATTTTAATGCATTTGATGCTCTAATGGGGTGGAAGCAGGA

GGCATTGCCACCAGTTGAAGTCCCTGCAGCAGCAAAATGGAAACTCAGAAGCAAACCCTT

CCAGCAAGTGGTACTCGATTATGACTATACATTCACAACACCTTATTGCGGCAGTGAGGC

AATTGACCTGAGTCAAGGAAAGATAATGTCTGAAAATGCATGTCCCAAACCCCATTGGGA

GGACTGTGATGAACAAATTGATTTGACTGCACTTTCAGCGAAGGAACCTATACTTTTTTA

TGACGAGGTGATCCTGTATGAAGATGAGTTAGCTGATAACGGAGTATCTCTGTTAACTGT

AAAAGTTAGAGTGATGCCCAGTTGTTTGTTTCTTCTACTTCGTTTTTGGCTTAGAGTAGA

TGGTGTACTGATGAGATTAAGGGATACTCGAGTTTATTGCGCTTTTAGTGATGACGATGC

TAAACCTGTTGTTCTTCGAGAGAGCTGCTGGAGAGAAGCAACCATCCAAAGTTTAACTGC

TAAAGGTTTCCCCTCTGATTCTGCTGCATACGGTGATCCGAACCACATCAGTCAGAGGCT

TCCCATAATCAAACATAAGACACAGAGGTTGAATATTTCTGGGTAACTTTGATTACTAGC

CCTGCTTGCACATTGTCACCCTACAAAACATCGTCTTTATGATAGAATTTATCTGACATG

AAAAATTACCATGTATGTTAGAGATGTTTGAAATTATGCTAGTTTTCCTTGCTTTTAACT

TTACCCACGGTGGAAGCCAGAATTTTCTGAAATACAATA
